# Fucoxanthin attenuates carbonyl stress and neuroinflammation by modulating MGO/RAGE/NF-κB axis in Aβ-induced models

**DOI:** 10.3389/fphar.2026.1811183

**Published:** 2026-06-15

**Authors:** Nayoung Lee, Kumju Youn, Huiyoung Kwon, Dong Hyun Kim, Chi-Tang Ho, Mira Jun

**Affiliations:** 1 Department of Health Sciences, The Graduate School of Dong-A University, Busan, Republic of Korea; 2 Department of Food Science and Nutrition, Dong-A University, Busan, Republic of Korea; 3 Center for Food and Bio Innovation, DAU G-LAMP Project Group, Dong-A University, Busan, Republic of Korea; 4 Department of Pharmacology, School of Medicine, Konkuk University, Seoul, Republic of Korea; 5 Department of Advanced Translational Medicine, School of Medicine, Konkuk University, Seoul, Republic of Korea; 6 Department of Food Science, Rutgers University, New Brunswick, NJ, United States

**Keywords:** Alzheimer’s disease, amyloid beta, carbonyl stress, fucoxanthin, MGO/RAGE/NF-κB axis, neuroinflammation

## Abstract

**Introduction:**

Amyloid-β (Aβ) accumulation is a central pathological feature of Alzheimer’s disease (AD) and a major driver of disease progression. Recent evidence suggests that carbonyl stress associated with Aβ plays a critical role in AD pathology by promoting neuroinflammation and neuronal damage. In particular, methylglyoxal (MGO), a highly reactive carbonyl compound, contributes to activation of the receptor for advanced glycation end products (RAGE) and NF-κB-dependent inflammatory signaling, leading to synaptic dysfunction. The present study investigated whether fucoxanthin, a marine-derived carotenoid, attenuates Aβ-induced carbonyl stress and inflammatory responses associated with MGO/RAGE/NF-κB-related signaling.

**Methods:**

PC12 neuronal cells were pretreated with fucoxanthin (0.1-5 μM) and exposed to aggregated Aβ_25-35_ (10 μM) to assess its effects on carbonyl stress–associated inflammatory signaling. In parallel, an Aβ_1-42_ intracerebroventricular injection mouse model was used to validate the *in vitro* findings. Mice were orally administered fucoxanthin (100 or 200 mg/kg/day) for 15 days and assessed for serum MGO levels, hippocampal RAGE/NF-κB activation, microglial activation, and synaptic marker expression.

**Results:**

Fucoxanthin significantly reduced the expression of pro-inflammatory mediators, including COX-2, iNOS, IL-1β, and TNF-α in Aβ-exposed neuronal cells. This anti-inflammatory effect was associated with inhibition of NF-κB nuclear translocation and downregulation of RAGE expression. Consistent with these *in vitro* findings, fucoxanthin treatment in Aβ_1-42_-injected mice alleviated systemic and hippocampal carbonyl stress, as evidenced by decreased serum MGO levels and suppression of hippocampal RAGE/NF-κB activation. These effects were accompanied by reduced microglial activation (Iba-1) across hippocampal subregions and significant restoration of both presynaptic and postsynaptic markers, indicating preservation of synaptic integrity.

**Conclusion:**

These findings demonstrate the neuroprotective role of fucoxanthin in mitigating Aβ-induced carbonyl stress by targeting the MGO/RAGE/NF-κB axis, thereby suppressing neuroinflammation and preserving synaptic integrity in Aβ-induced cellular and mouse models. Fucoxanthin emerges as a promising pharmacological candidate targeting carbonyl stress–associated mechanisms in AD.

## 1 Introduction

Alzheimer’s disease (AD) is the most common form of dementia, affecting at least 55 million people worldwide and accounting for an estimated 60%–80% of all dementia cases (Alzheimer’s disease facts and figures, 2024). The pathological hallmarks of AD include abnormal amyloid plaque formation and neurofibrillary tangles deposition, which contribute to progressive neurodegeneration (Bloom, 2014). Aβ peptides, the primary constituents of amyloid plaques, serve as key pathogenic mediators by inducing oxidative stress, neuroinflammation and apoptosis, thereby accelerating AD progression (Azargoonjahromi, 2024; Zhang et al., 2024). Emerging evidence further indicates that Aβ contributes to the development of carbonyl stress, which is increasingly recognized as a critical factor in AD pathogenesis (Jana et al., 2016).

Aβ accumulation induces oxidative stress, which, in turn, promotes the formation of reactive carbonyl species (RCS), including glyoxal, 4-hydroxy-2-nonenal (4-HNE), and methylglyoxal (MGO), thereby intensifying carbonyl stress. Although MGO is primarily a by-product of glucose metabolism, its excessive production in the AD brain has been attributed to Aβ-induced disruptions in neuronal energy metabolism. Specifically, Aβ disrupts glycolysis and mitochondrial function, resulting in the accumulation of glycolytic intermediates such as triose phosphates, key precursors of MGO, while concurrent impaired glucose uptake in the AD brain further contributes to this accumulation (Zhang et al., 2021). Importantly, both preclinical and clinical studies have demonstrated that reduced glycolytic flux is closely associated with the progression of amyloid and tau pathology, highlighting the pathological relevance of impaired glucose metabolism in AD (Vlassenko et al., 2018). Moreover, MGO has been shown to facilitate Aβ aggregation, establishing a self-perpetuating pathological cycle (Vlassenko et al., 2018; Goyal et al., 2020). Clinical studies further support the significance of carbonyl stress, reporting that elevated plasma MGO levels are correlated with cognitive decline in AD patients (Beeri et al., 2011; Angeloni et al., 2014).

Aβ-induced MGO accumulation upregulates the expression of the receptor for advanced glycation end-products (RAGE), a multi-ligand receptor primarily involved in pro-inflammatory signaling. RAGE interacts with AGEs, S100 proteins, and Aβ, triggering intracellular signaling cascades that lead to sustained neuroinflammation. Upon activation, RAGE initiates translocation of nuclear factor kappa-light-chain-enhancer of activated B cells (NF-κB) to the nucleus, where it alters gene expression and drives a chronic inflammatory response. This response enhances the transcription of pro-inflammatory enzymes and cytokines, including inducible nitric oxide synthase (iNOS), cyclooxygenase-2 (COX-2), tumor necrosis factor-alpha (TNF-α), and interleukin-1β (IL-1β), all of which contribute to neuronal damage (Bierhaus and Nawroth, 2009; Lue et al., 2001). Furthermore, RAGE/NF-κB activation contributes to mitochondrial dysfunction and increased reactive oxygen species (ROS) production, thereby intensifying redox imbalance and cellular stress (Bierhaus and Nawroth, 2009). These processes underscore the pivotal role of the MGO/RAGE/NF-κB axis in linking metabolic dysfunction to neuroinflammation and oxidative stress, ultimately driving AD progression. These observations are further supported by previous AD-related studies linking MGO/AGE-RAGE signaling to key pathological features of AD (Batkulwar et al., 2018; Pucci et al., 2021; Moreira et al., 2022). Batkulwar et al. demonstrated that AGE-RAGE signaling influences amyloidogenic APP processing and tau phosphorylation, suggesting a direct contribution of MGO-derived glycation to core AD-associated proteopathic alterations (Batkulwar et al., 2018). In addition, studies using STZ- or MGO-induced AD models, as well as analyses of AD patient brain tissues, have reported persistent activation of the MGO/RAGE signaling axis accompanied by increased Aβ burden, tau pathology, oxidative stress, and neuroinflammatory responses (Pucci et al., 2021). Persistent activation of the MGO/RAGE-associated signaling axis has also been associated with sustained microglial activation, enhanced oxidative damage, and progressive neurodegenerative alterations associated with AD pathology under streptozotocin-induced conditions (Moreira et al., 2022).

Given the pivotal role of neuroinflammation in AD, microglia activation has emerged as a critical pathological component. Microglia, the resident immune cells of the central nervous system, play a crucial role in clearing cellular debris, modulating synaptic plasticity, and responding to neuroinflammatory stimuli (Norris and Kipnis, 2019; Samsuzzaman et al., 2024). Positioned near cerebral microvessels, they continuously monitor the extracellular environment for potential threats. Accumulating evidence has demonstrated that MGO promotes microglial activation through the RAGE/NF-κB pathway, driving their polarization toward a pro-inflammatory phenotype and sustaining neuroinflammation and neuronal injury (Fan et al., 2020; Schalkwijk and Stehouwer, 2020; Zhang et al., 2022). Notably, RAGE is highly co-localized with Iba1-positive microglia in the AD brain, linking carbonyl stress to persistent neuroinflammation and synaptic dysfunction (Wetzels et al., 2019).

Marine-derived bioactive compounds have gained increasing interest for their ability to modulate oxidative stress, inflammation, and carbonyl stress (Liu and Gu, 2012; Menaa et al., 2021). Fucoxanthin, a carotenoid abundant in brown algae, has demonstrated potent antioxidant, anti-inflammatory, and neuroprotective effects in various neurodegenerative models (Miyashita et al., 2020). Structurally, fucoxanthin possesses an allenic bond, carbonyl group, and epoxide moiety, which contribute to its broad range of biological activities (Peng et al., 2011). Previous studies, including our own, have shown that fucoxanthin reduces oxidative damage, inhibits neuronal apoptosis, and suppresses inflammatory signaling pathways, making it a promising candidate for neuroprotection (Lin et al., 2017; Hu et al., 2018; Jiang et al., 2019; Lee et al., 2023). However, the role of fucoxanthin in modulating carbonyl stress-mediated mechanisms associated with Aβ pathology remains insufficiently understood. The present study aimed to investigate the effects of fucoxanthin on the MGO/RAGE/NF-κB signaling pathway in Aβ-treated cellular and mouse models by assessing MGO burden, inflammatory marker expression, and synaptic protein levels as indicators of its preventive potential.

## 2 Materials and methods

### 2.1 Reagents and chemicals

Fucoxanthin (purity ≥98%) was purchased from Aktin Chemicals, Inc. (Chengdu, China). Aβ_1-42_ (purity ≥95%) was purchased from Anaspec (Fremont, CA, United States). PC12 cells were supplied from American Type Culture Collection (ATCC, Rockville, MD, United States). Aβ_25-35_ (purity >95%) was obtained from Genscript (Piscataway, NJ, United States). Roswell Park Memorial Institute (RPMI) cell culture medium, fetal bovine serum (FBS), trypsin-EDTA, horse serum, and penicillin-streptomycin were purchased from Hyclone Laboratories (Logan, UT, United States). RPMI 1640 phenol red free medium was supplied by Gibco BRL (Grand Island, NY, United States). Phosphate-buffered saline (PBS) was purchased from Welgene (Gyeongsan, South Korea). N_2_ supplement was obtained from Invitrogen (Carlsbad, CA, United States). The MGO enzyme-linked immunosorbent assay (ELISA) kit was obtained from Abcam (Cambridge, MA, United States). Paraformaldehyde (PFA) was obtained from Biosesang (Seongnam, South Korea). Optimum cutting temperature (OCT) compound was purchased from Leica Biosystems (Wetzlar, Germany). Avidin-biotin peroxidase complex (ABC), normal goat serum, and 3, 3′-diamino-benzidine (DAB) were obtained from Vector Laboratories, Inc. (Newark, CA, United States). dimethyl sulfoxide (DMSO), 3-(4,5-dimethylthiazol-2-yl)-2,5-diphenyl-tetrazolium (MTT), Triton X-100, Tween 80, and radioimmunoprecipitation assay (RIPA) buffer were obtained from Sigma-Aldrich (St. Louis, MO, United States). Protease and phosphatase inhibitor cocktails, NE-PERTM Nuclear and Cytoplasmic Extraction Reagents and BCA protein assay kits were obtained from Thermo Fisher Scientific (Waltham, MA, United States). SDS-PAGE loading buffer (5×) was purchased from Lab-Pharm-Service (LPS) solution (Daejeon, South Korea). Polyvinylidene fluoride (PVDF) was purchased from Millipore Corporation (Billerica, MD, United States). The enhanced chemiluminescence (ECL) detection reagent was purchased from Advansta Inc. (San Jose, CA, United States). The primary antibodies used for Western blot and immunohistochemistry are listed in Table 1. Secondary antibodies were obtained from Bethyl Laboratories, Inc. (Montgomery, TX, United States).

**TABLE 1:** Characteristics of primary antibodies.

Antibody	Dilution	Species	Source (catalog no.)
RAGE	1:1000	Rabbit	Cell signaling (#42544)
p-NF-κB p65	1:1000	Rabbit	Cell signaling (#3033)
NF-κB p65	1:1000	Mouse	Santa cruz (sc-8008)
p-I-κB	1:2000	Rabbit	Cell signaling (#2859)
I-κB	1:2000	Rabbit	Cell signaling (#9242)
COX-2	1:2000	Rabbit	GeneTex (GTX60935)
iNOS	1:1000	Rabbit	Cusabio Co. Ltd. (CSB-PA003464)
IL-1β	1:1000	Rabbit	GeneTex (GTX74034)
TNF-α	1:1000	Rabbit	Cell signaling (#11948)
PSD-95	1:3000	Rabbit	Cell signaling (#3450)
Synaptophysin	1:3000	Rabbit	Cell signaling (#36406)
β-actin	1:10000	Mouse	Santa cruz (sc-47778)
PCNA	1:3000	Rabbit	GeneTex (GTX100539)
Iba-1	1:500	Rabbit	Abcam (ab178847)

### 2.2 Cell culture and Aβ_25-35_ treatment

PC12 cells were maintained in RPMI 1640 supplemented with 10% horse serum, 5% FBS, and 100 U/mL penicillin-streptomycin at 37 °C in a humidified 5% CO2 incubator. For different experimental purposes, cells were seeded into plates of various sizes and incubated overnight and then replaced with fresh N2 medium. For Western blotting, the cells were plated in 6-well plates at a density of 1 × 106 cells/well and subsequently treated with fucoxanthin and Aβ_25-35_ as described below.

The Aβ_25-35_ peptides, a neurotoxic fragment of the full-length Aβ protein, was selected for *in vitro* studies due to its rapid aggregation properties and well-documented ability to induce neurotoxicity in neuronal cultures (Varadarajan et al., 2001). Aβ_25-35_ was dissolved in PBS at concentration of 1 mM to prepare a stock solution and was incubated at 37 °C for 2 days to induce aggregation. The stock solution was stored at −70 °C prior to use and diluted to 10 μM. The cells were pretreated with fucoxanthin (0.1, 1, and 5 μM) or vehicles (0.1% DMSO) for 1 h, followed by exposure to 10 μM Aβ_25-35_.

### 2.3 Cell viability assay

The cells were plated in 96-well plates at the density of 1 × 10^4^ cells/well and treated with various concentrations of fucoxanthin for 24 h. Following treatment MTT solution (5 mg/mL, 10 µL) was added to each well and incubated for 2 h at 37 °C in the dark. After incubation, the medium was discarded, and DMSO was added to solubilize the purple formazan crystals. The absorbance was measured at 570 nm using a Synergy H1 multi-mode reader (Biotek Instruments, Inc. Winooski, VT, United States).

### 2.4 Animals and Aβ_1-42_ injection

Specific-pathogen-free male ICR mice (6 weeks old, 25 ± 5 g) were purchased from Samtako (Osan, South Korea). All animal experiments were conducted in accordance with guidelines approved by the Institutional Animal Care and Use Committee of Dong-A University (DIACUC-approve-23-26). Mice were randomly assigned to four groups before Aβ_1-42_ injection: Sham, Aβ + vehicle, Aβ + fucoxanthin 100 mg/kg, and Aβ + fucoxanthin 200 mg/kg. The sample size was determined based on previous comparable *in vivo* mouse model studies and ethical considerations to minimize animal use (Cyr et al., 2024; Kaur et al., 2024; Kim et al., 2025; Lee et al., 2025; Maddila et al., 2026). Mice were acclimated for 6 days before experimentation and maintained under standard conditions (23 °C ± 2 °C, 50% ± 10% relative humidity, 12 h light/dark cycle) with *ad libitum* access to water and food.

To investigate the direct effects of Aβ on carbonyl stress and inflammatory signaling, an acute intracerebroventricular Aβ injection model was employed in young animals under temporally controlled conditions(Kim et al., 2016; Vande Vyver et al., 2023). Specifically, Aβ_1-42_ was used *in vivo* studies due to its ability to form fibrillar aggregates similar to amyloid plaques observed in AD (Xiao et al., 2015). Aβ_1-42_ powder (1 mg) was dissolved in PBS at 1 mg/mL and incubated for 72 h at 37 °C with gentle shaking. Aggregated Aβ_1-42_ (5 μL/mouse) was injected into the third ventricle using a Hamilton microsyringe. The stereotaxic coordinates used for injection were as follows: anterior-posterior (AP): - 2.00 mm; medial-lateral (ML), 0 mm; dorso-ventral (DV), - 2.00 mm.

### 2.5 Sample treatment

Following Aβ_1-42_ injection, mice in the treatment groups received fucoxanthin at doses of 100 and 200 mg/kg/day for 15 consecutive days. Fucoxanthin was suspended in 10% Tween 80 solution and administered once daily by oral gavage. The sham and Aβ + vehicle groups received an equivalent volume of 10% Tween 80 solution by oral gavage. Then, all animals were perfused with PBS, and the brains were extracted and dissected into two sagittal halves. One hemisphere was used for immunohistochemistry (IHC), and the other hemisphere was used for Western blot analysis.

### 2.6 Serum MGO measurement

Whole blood was collected post-sacrifice, centrifuged at 3,000 rpm for 10 min, and the serum was separated and stored at −80 °C until further analysis. MGO levels in mouse serum were quantified using an ELISA kit according to the manufacturer’s instructions (Abcam). The absorbance of the standards and samples was measured at a wavelength of 450 nm using a Synergy H1 multimode reader (Biotek Instruments, Inc.).

### 2.7 IHC analysis

Brain tissues were fixed in 4% PFA overnight at 4 °C, followed by immersion in 30% sucrose until the tissues completely sank. Afterward, the brains were embedded in OCT compound, and 30 µm-thick serial sections were prepared using a freezing cryotome (Microm HM525, Leica, Nussloch, Germany). The sectioned brain tissues were stored in cryoprotection storage solution (10% 0.2 M PB, 30% ethylene glycol, and 30% glycerin) at 4 °C.

Immunohistochemical staining was performed using the free-floating and ABC method. Briefly, selected sections were rinsed in PBS and blocked with 1% H2O2 in methanol to inactivate peroxidase activity. After washing, the sections were blocked with blocking buffer (3% normal goat serum and 0.3% Triton X-100 in PBS) to prevent nonspecific antigen binding, followed by primary antibody incubation with Iba-1 (2% normal goat serum and 0.3% Triton X-100). The following day, the sections were exposed to biotinylated secondary antibody (1:200 dilution, 1% serum, and 0.3% Triton X-100) and ABC. The signal was visualized using DAB for 1 min and mounted onto silane-coated slides. After dehydration through graded ethanol (70%, 80%, 90%, 95% and 100%) and xylene treatment, samples were analyzed.

Because the hippocampus is particularly vulnerable to Aβ-related synaptic dysfunction and neuroinflammatory pathology in AD, Iba-1 immunoreactivity was quantitatively assessed in the CA1, CA3, and DG regions of the hippocampus (Zhang et al., 2022; Sanchez-Rodriguez et al., 2024; Behroozi et al., 2026). For each animal, two to three sections at equivalent anatomical levels were analyzed. Images were captured at ×100 magnification (scale bar = 200 μm) and converted to 16-bit grayscale. The Iba-1-positive area was measured using ImageJ software (v. 1.52a, NIH, Bethesda, MD, United States) and expressed as a percentage of the total image area (Iba-1-positive area/total area × 100). All quantification was performed by an investigator blinded to the experimental group assignments.

### 2.8 Western blot analysis

PC12 cells or hippocampal tissue lysates were lysed with ice-cold RIPA buffer containing protease and phosphatase inhibitor cocktails and centrifuged at 12,000 rpm for 30 min at 4 °C. The supernatant was collected, and protein concentration was measured with a BCA protein assay kit. Equal amounts of protein (20 µg) were mixed with a 5× loading buffer, denatured at 95 °C for 5 min, and separated on 9%–12% SDS-PAGE gels. After electrophoresis, the proteins were transferred to PVDF membranes. Blots were subsequently incubated with 5% skimmed milk for 1 h at room temperature and then incubated with various specific primary antibodies overnight at 4 °C (Table 1). The membranes were washed with Tris-buffered saline containing Tween 20 (TBST) and further incubated with horseradish peroxidase (HRP)-conjugated secondary antibodies for 7-10 min at room temperature with shaking. Protein signals were detected using an ECL detection reagent and visualized through Atto EZ-capture (Tokyo, Japan). Band intensities were measured using ImageJ software (v. 1.52a, NIH, Bethesda, MD, United States). Target protein levels in whole-cell and cytoplasmic fractions were normalized to β-actin, whereas those in the nuclear fraction were normalized to PCNA. Phosphorylated protein levels were normalized to their corresponding total protein levels. The normalized values were then expressed as a percentage relative to the control or sham group, which was set to 100%.

### 2.9 Statistical analysis

All experimental data are presented as mean ± standard deviation (SD) for *in vitro* assays and as mean ± standard error of the mean (SEM) for *in vivo* assays. Prior to statistical analysis, the normality of data distribution for all continuous variables was assessed using the Shapiro–Wilk test, and homogeneity of variances across groups was evaluated using the Brown–Forsythe test. When both assumptions were met (p > 0.05), Student’s t-test was used for comparisons between two groups, and one-way ANOVA followed by Tukey’s *post hoc* test was used for multiple group comparisons. Otherwise, the Mann–Whitney U test was applied for two-group comparisons, and the Kruskal–Wallis test followed by Dunn’s *post hoc* test was used for multiple group comparisons. In addition, a sensitivity analysis was performed using G*Power version 3.1.9.7 under the current experimental design (4 groups, total n = 20, α = 0.05) to estimate the detectable effect size under the present sample conditions. All statistical analyses were conducted using Prism version 8.0.2 (GraphPad Software, San Diego, CA, United States). A p-value <0.05 was considered statistically significant.

## 3 Results

### 3.1 Fucoxanthin alleviates Aβ_25-35_-induced cytotoxicity in PC12 cells

**FIGURE 1:**
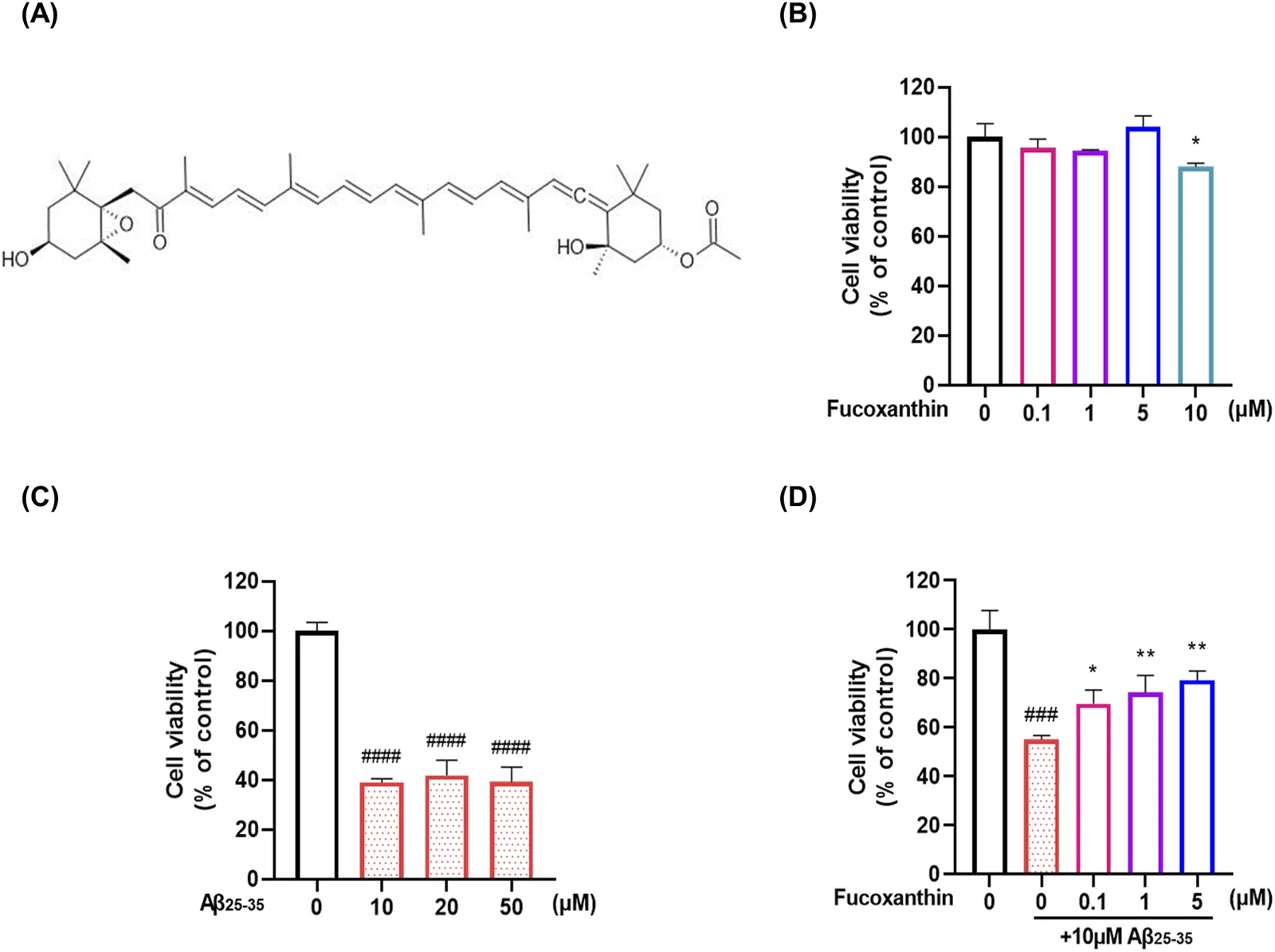
Protective effects of fucoxanthin against Aβ_25-35_-induced cytotoxicity in PC12 cells. **(A)** Chemical structure of fucoxanthin. Cell viability of PC12 cells treated with various concentrations of **(B)** fucoxanthin (0.1-5 µM) and **(C)** Aβ_25-35_ (10–50 µM) for 24 h. **(D)** The protective effect of fucoxanthin against Aβ_25-35_ toxicity. The cells were incubated with the sample for 1 h, followed by incubation with Aβ_25-35_ for another 24 h. Cell viability was expressed as a percentage relative to the control group, which was set to 100%. Data are expressed as mean ± SD and represent three independent experiments with three replications per experiment. ^###^P < 0.001 and ^####^P < 0.0001 vs. control group; *P < 0.05 and **P < 0.01 vs. Aβ_25-35_-treated group.

The chemical structure of fucoxanthin is shown in Figure 1A. To evaluate its neuroprotective potential, PC12 cells were treated with Aβ_25-35_, a neurotoxic fragment widely used to model Aβ-induced cellular damage. Fucoxanthin at concentrations of 0.1 to 5 µM exhibited no cytotoxicity in PC12 cells (Figure 1B). However, Aβ_25-35_ exposure (10 μM) significantly reduced cell viability compared to the control group (P < 0.0001, Figure 1C). Notably, fucoxanthin restored cell viability in a dose-dependent manner, mitigating Aβ-induced cytotoxicity (Figure 1D).

### 3.2 Fucoxanthin reduces carbonyl stress-associated RAGE/NF-κB-related changes in Aβ_25–35_-treated PC12 cells

**FIGURE 2:**
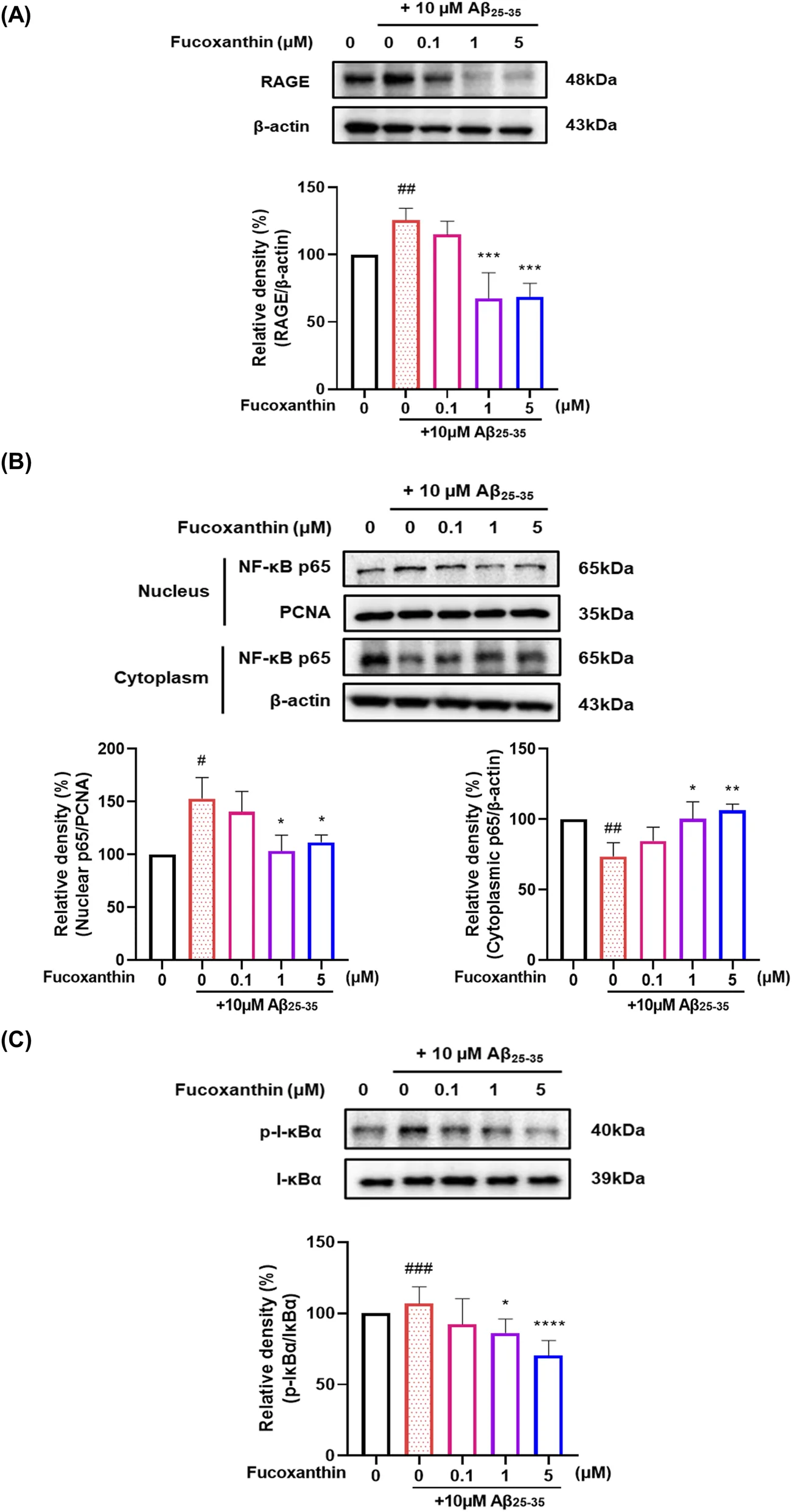
Inhibitory effects of fucoxanthin on RAGE expression and NF-κB activation in Aβ_25-35_-treated PC12 cells. Immunoblot analysis and quantification of **(A)** RAGE, **(B)** NF-κB p65 nuclear and cytoplasmic fractions, and **(C)** p-I-κB levels. Nuclear p65 was normalized to PCNA, cytoplasmic p65 and RAGE were normalized to β-actin, and p-IκB was normalized to total IκB. The cells were incubated with fucoxanthin for 1 h, followed by incubation with Aβ_25-35_ for another 4 h. The normalized values were expressed as percentages relative to the control group, which was set to 100%. Data are expressed as mean ± SD and represent three independent experiments with three replications per experiment. ^#^P < 0.05, ^##^P < 0.01, and ^###^P < 0.001 vs. control group; *P < 0.05, **P < 0.01, ***P < 0.001, and ****P < 0.0001 vs. Aβ_25-35_-treated group.

RAGE plays a central role in carbonyl stress-induced neuroinflammation by facilitating NF-κB signaling. In Aβ_25-35_-treated PC12 cells, RAGE expression was significantly upregulated compared to the control group (125.87% ± 8.52%, P < 0.01, Figure 2A. This increase was effectively suppressed by fucoxanthin co-treatment at both 1 and 5 μM, which reduced RAGE expression to 67.51% ± 19.04% and 68.8% ± 10.02% of the control group, respectively (P < 0.001).

Consistent with RAGE activation, Aβ_25-35_ exposure enhanced NF-κB nuclear translocation, as evidenced by increased nuclear NF-κB levels 152.92% ± 20.02% of the control group, P < 0.05, Figure 2B) and a corresponding reduction in cytoplasmic levels (73.74% ± 9.73% of the control group, P < 0.01). Fucoxanthin treatment was accompanied by reduced nuclear NF-κB accumulation and restoration of cytoplasmic NF-κB levels toward baseline (P < 0.05 and P < 0.01, respectively).

To further substantiate NF-κB pathway activation, phosphorylated IκB (p-IκB) levels were analyzed. A marked elevation in p-IκB expression was observed in response to Aβ_25-35_ treatment (P < 0.001, Figure 2C). This elevation was significantly suppressed by fucoxanthin in a dose-dependent manner (P < 0.05 and P < 0.0001, respectively).

### 3.3 Fucoxanthin downregulates NF-κB-dependent inflammatory mediators in PC12 cells

**FIGURE 3:**
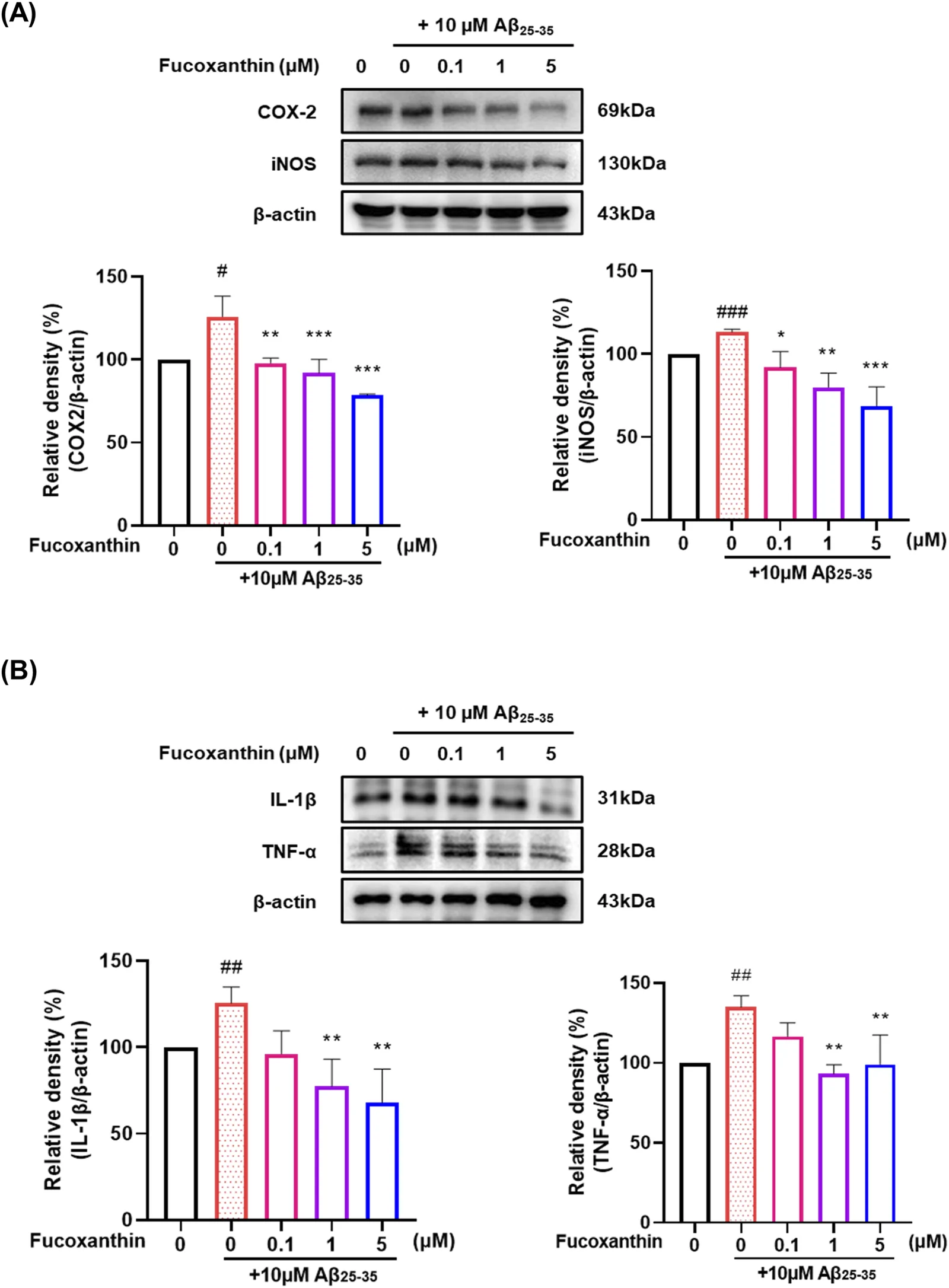
Effects of fucoxanthin on pro-inflammatory mediators in Aβ_25-35_-treated PC12 cells. Immunoblot analysis and quantification of **(A)** COX-2 and iNOS, and **(B)** IL-1β and TNF-α expression. β-actin was used as a normalizing control. The cells were incubated with fucoxanthin for 1 h, followed by incubation with Aβ_25-35_ for another 24 h. The normalized values were expressed as percentages relative to the control group, which was set to 100%. Data are expressed as mean ± SD and represent three independent experiments with three replications per experiment. ^#^P < 0.05, ^##^P < 0.01, and ^###^P < 0.001 vs. control group; *P < 0.05, **P < 0.01, and ***P < 0.001 vs. Aβ_25-35_-treated group.

To assess whether NF-κB inhibition led to reduced inflammatory responses, the expression levels of the pro-inflammatory enzymes COX-2 and iNOS, as well as the cytokines IL-1β and TNF-α, were analyzed. Aβ_25-35_ exposure significantly upregulated COX-2 and iNOS levels (P < 0.05 and P < 0.001, respectively, Figure 3A), whereas fucoxanthin at all tested concentrations (0.1-5 μM) effectively downregulated both enzymes to near-control values. Similarly, IL-1β and TNF-α levels were markedly increased following Aβ_25-35_ exposure (P < 0.01, Figure 3B), and were significantly attenuated by fucoxanthin at 1 and 5 μM (P < 0.01).

### 3.4 Fucoxanthin attenuates carbonyl stress-associated RAGE/NF-κB signaling and associated synaptic alterations in Aβ_1-42_-injected mice

**FIGURE 4:**
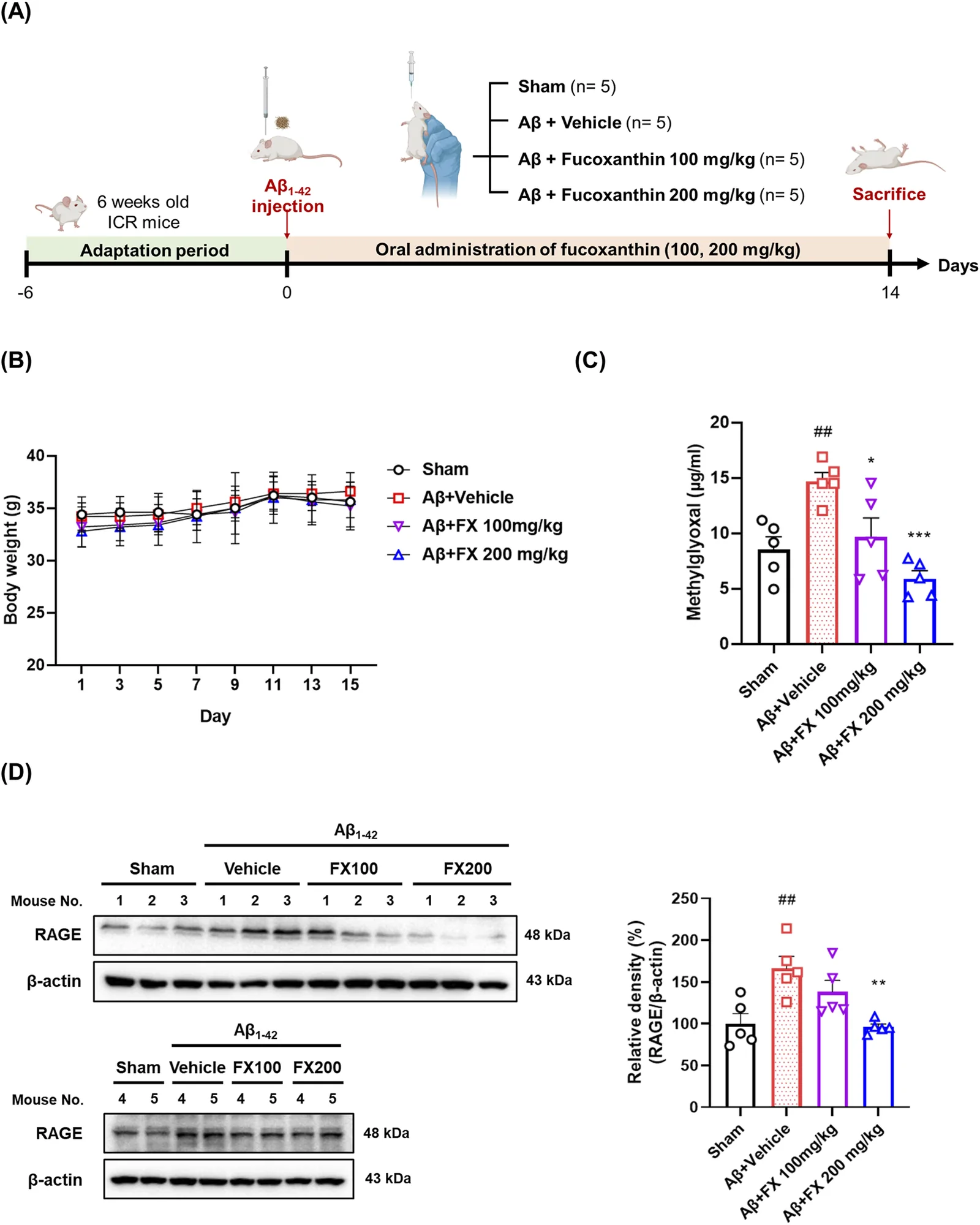
Effects of fucoxanthin on carbonyl stress and neurotoxicity in Aβ_1-42_-injected mice. **(A)** Experimental timeline for Aβ_1-42_ injection and fucoxanthin administration. **(B)** Changes in body weight during the experimental period. **(C)** Serum MGO levels measured using ELISA. Immunoblot bands and quantification of **(D)** RAGE expression in hippocampal tissue. β-actin was used as a normalizing control. The normalized RAGE values were expressed as percentages relative to the sham group, which was set to 100%. Western blot images from all animals are presented across two separate gels. Data are expressed as mean ± SEM (n = 5 per group) and represent three independent experiments with three replications in each experiment. ^##^P < 0.01 vs. sham group; *P < 0.05, **P < 0.01 and ***P < 0.001 vs. Aβ_1-42_-injected group. Figure 4A was Created in BioRender. Lee, N. (2026) https://BioRender.com/fouktlk.

To validate the *in vitro* findings, mice were intracerebroventricularly injected with Aβ_1-42_ and subsequently administered oral fucoxanthin (100 or 200 mg/kg) for 15 days (Figure 4A). No significant changes in body weight were observed among the groups during the experimental period (Figure 4B). Serum MGO levels, a key marker of carbonyl stress, were markedly increased in Aβ_1-42_-injected mice (14.7 ± 1.78 µg/mL, P < 0.01, Figure 4C). Fucoxanthin administration reduced MGO levels in a dose-dependent manner, with the 200 mg/kg group showing a decrease to 5.92 ± 1.59 µg/mL (P < 0.001).

**FIGURE 5:**
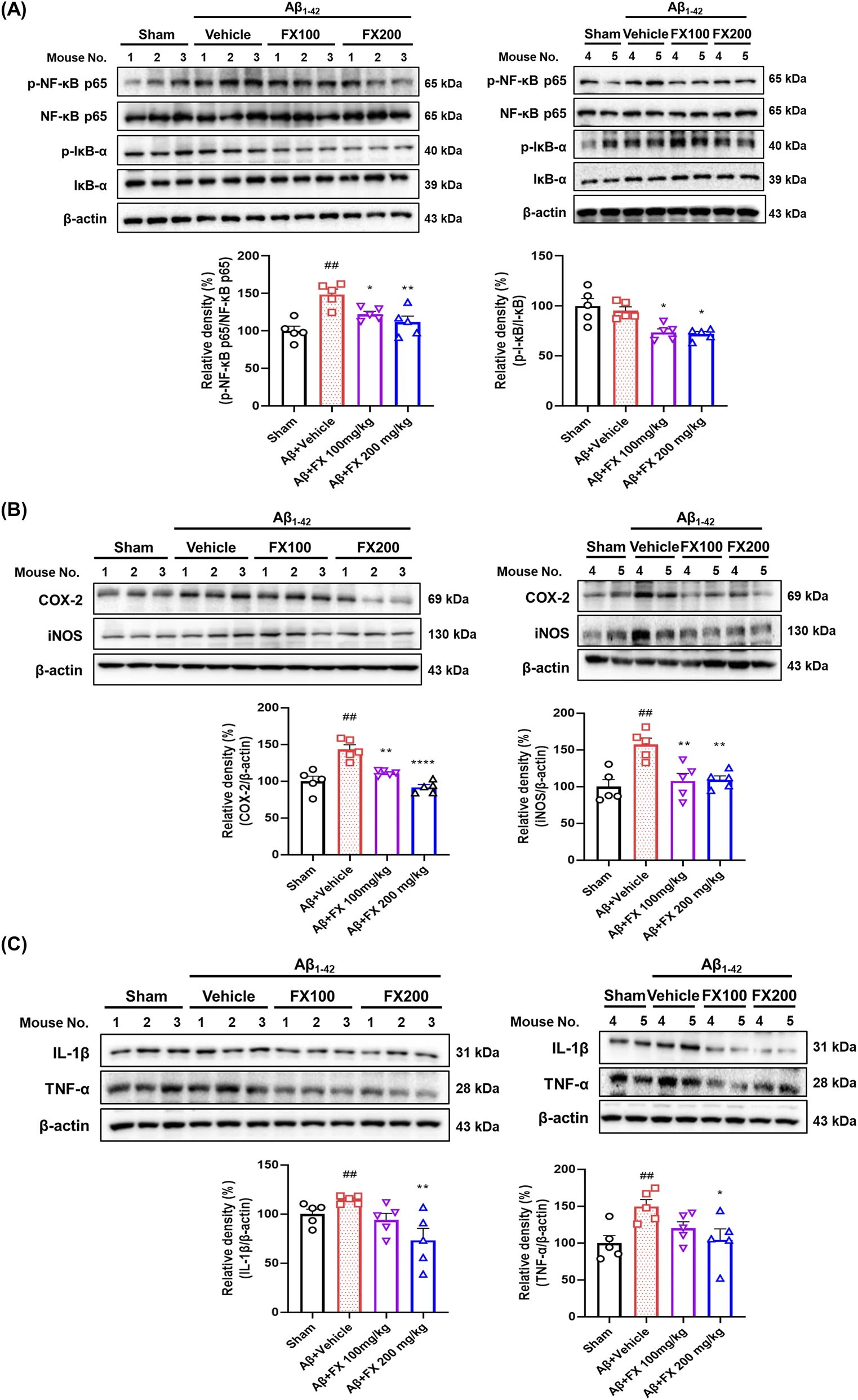
Inhibitory effects of fucoxanthin on NF-κB activation in Aβ_1-42_-injected mice. Immunoblot bands and quantification of **(A)** p-NF-κB and p-I-κB, **(B)** COX-2 and iNOS, and **(C)** IL-1β and TNF-α expression in hippocampal tissue. p-NF-κB p65 and p-IκB were normalized to their corresponding total proteins, whereas COX-2, iNOS, IL-1β, and TNF-α were normalized to β-actin. The normalized values were expressed as percentages relative to the sham group, which was set to 100%. Western blot images from all animals are presented across two separate gels. Data are expressed as mean ± SEM (n = 5 per group) and represent three independent experiments with three replications in each experiment. ^##^P < 0.01 vs. sham group; *P < 0.05, **P < 0.01, and ****P < 0.0001 vs. Aβ_1-42_-injected group.

In parallel, hippocampal RAGE expression was substantially upregulated in the Aβ_1-42_-injected brains, reaching 166.39% ± 32.13% of the sham group (P < 0.01, Figure 4D), while fucoxanthin treatment (200 mg/kg) normalized RAGE levels to those of the sham group (96.34% ± 7.83% of the sham group, P < 0.01). Downstream signaling analysis revealed that Aβ_1-42_ injection increased phosphorylated NF-κB p65 levels to 148.98% ± 15.55% of the sham group (P < 0.01, Figure 5A). Fucoxanthin administration significantly suppressed p-NF-κB p65 expression at all tested doses, accompanied by a corresponding, dose-dependent reduction in p-IκB levels.

To further assess the inflammatory consequences of NF-κB modulation, hippocampal levels of pro-inflammatory mediators were evaluated. Aβ_1-42_ injection led to pronounced upregulation of COX-2, iNOS, IL-1β, and TNF-α in the hippocampus (P < 0.01, Figures 5B,C). Fucoxanthin treatment significantly reduced COX-2 and iNOS expression across all tested doses, while reductions in IL-1β and TNF-α were observed at 200 mg/kg.

**FIGURE 6:**
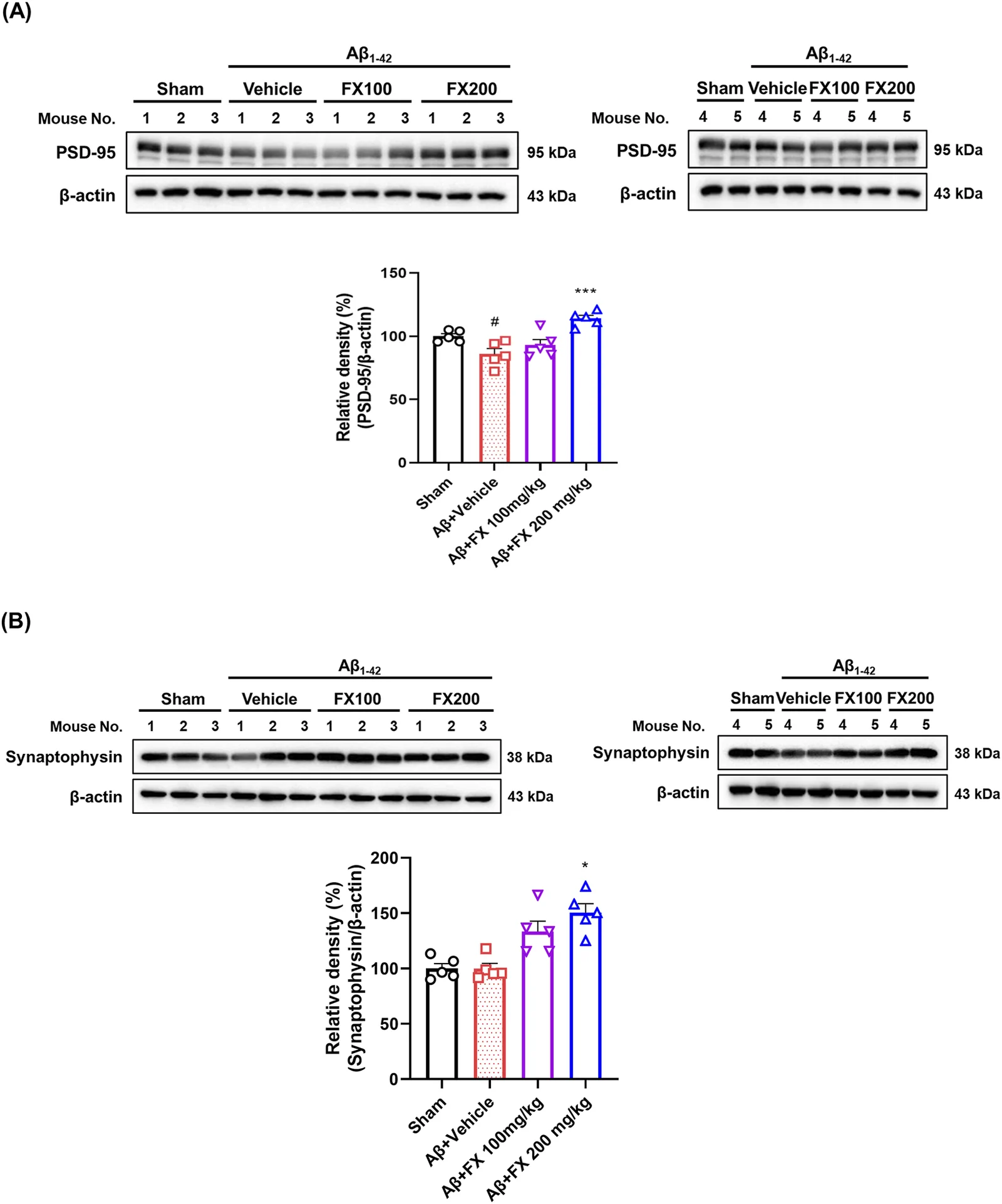
Protective effects of fucoxanthin on synaptic protein expression in Aβ_1-42_-injected mice. Immunoblot bands and quantification of **(A)** PSD-95 and **(B)** synaptophysin expression in hippocampal tissue. β-actin was used as a normalizing control. The normalized values were expressed as percentages relative to the sham group, which was set to 100%. Western blot images from all animals are presented across two separate gels. Data are expressed as mean ± SEM (n = 5 per group) and represent three independent experiments with three replications in each experiment. ^#^P < 0.05 vs. sham group; *P < 0.05 and ***P < 0.001 vs. Aβ_1-42_-injected group.

In addition to inflammatory signaling, synaptic markers were examined. The decline in PSD-95 expression observed in Aβ_1-42_-injected mice was significantly ameliorated by fucoxanthin (200 mg/kg, P < 0.001, Figure 6A). In contrast, synaptophysin expression was not significantly altered by Aβ_1-42_ injection alone (99.91% ± 10.35% of the sham group, P > 0.05, Figure 6B). Fucoxanthin administration significantly increased synaptophysin levels in a dose-dependent manner, compared with the Aβ group, reaching 150.58% ± 18.02% at 200 mg/kg (P < 0.05).

### 3.5 Fucoxanthin suppresses microglial activation in Aβ_1-42_-injected mice

**FIGURE 7:**
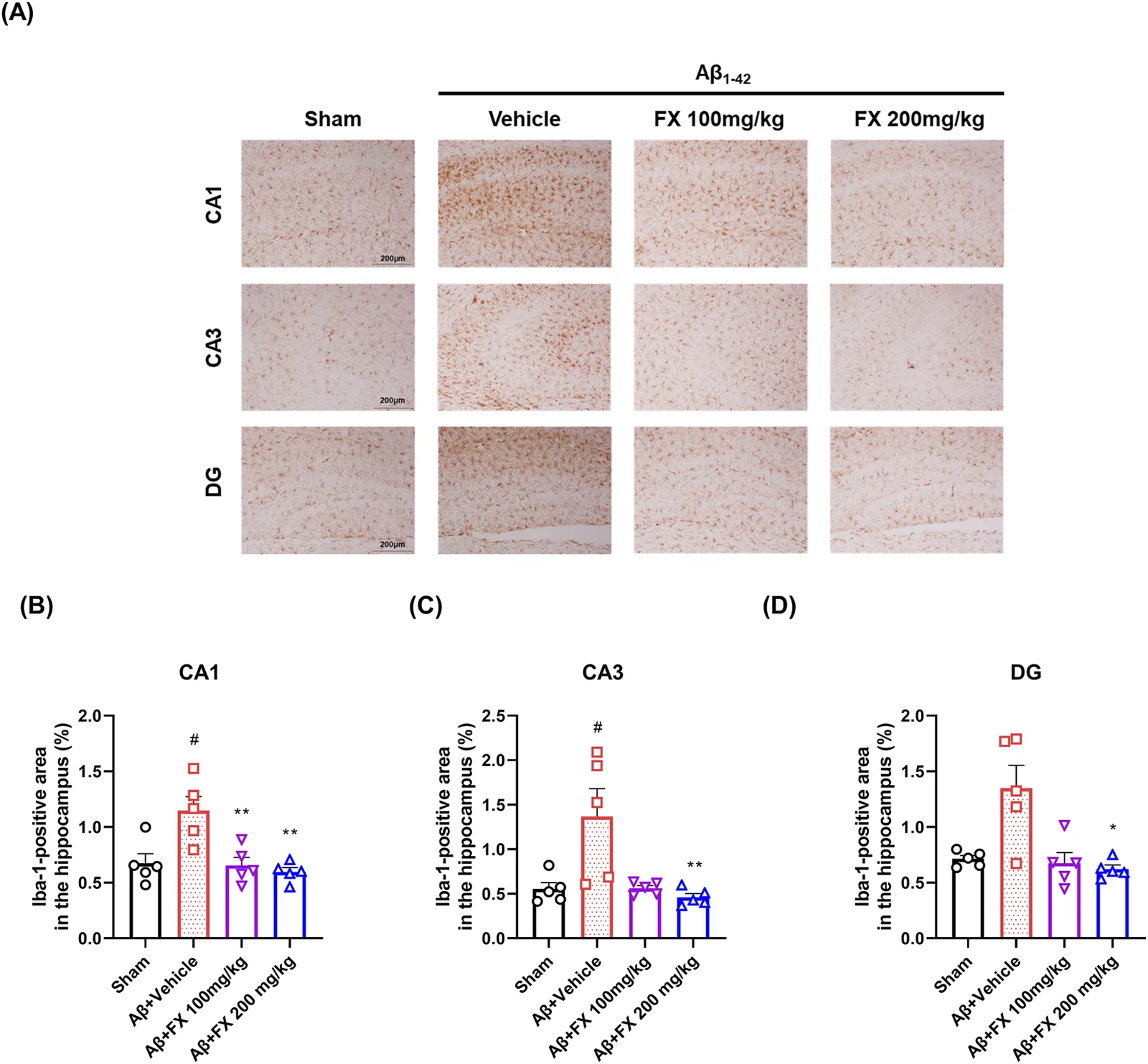
Inhibitory effects of fucoxanthin on microglial activation through carbonyl stress reduction in Aβ_1-42_-injected mice. **(A)** Representative immunohistochemical staining images of Iba-1-positive microglia in the CA1, CA3, and DG subregions of the hippocampus. Scale bar = 200 μm. **(B–D)** Quantification of Iba-1-positive area in the CA1, CA3, and DG regions. Data are expressed as mean ± SEM (n = 5 per group). ^#^P < 0.05, vs. sham group; *P < 0.05 and **P < 0.01 vs. Aβ_1-42_-injected group.

To explore the association between carbonyl stress and neuroinflammation, Iba-1-positive microglial density was quantified in hippocampal subregions cornu ammonis 1 (CA1), cornu ammonis 3 (CA3), and dentate gyrus (DG) regions of the hippocampus. As shown in Figure 7, Aβ_1-42_-injection significantly increased the percentage of Iba-1-positive area in CA1 (1.15% ± 0.17%) and CA3 (1.37% ± 0.18%) compared to the sham group (P < 0.05). Both fucoxanthin 100 and 200 mg/kg significantly reduced Iba-1-positive area in CA1 compared to the Aβ+vehicle group (P < 0.01), while only fucoxanthin 200 mg/kg showed significant effects in CA3 (P < 0.01) and DG (P < 0.05).

## 4 Discussion

Recent advances in AD research have increasingly recognized carbonyl stress as an additional pathogenic mechanism alongside oxidative stress and neuroinflammation, underscoring its contribution to disease progression (Rahmadi et al., 2011; Jana et al., 2016). Among various reactive carbonyl species, MGO has emerged as a pivotal mediator due to its involvement in both protein glycation and the activation of inflammatory signaling pathways (Zhang et al., 2021). Elevated MGO levels in the AD brain not only exacerbate Aβ aggregation but also stimulate RAGE, triggering downstream NF-κB signaling and amplifying transcription of pro-inflammatory mediators (Bierhaus and Nawroth, 2009; Vlassenko et al., 2018; Goyal et al., 2020). This MGO/RAGE/NF-κB axis serves as a critical interface between metabolic dysfunction and sustained neuroinflammation, ultimately contributing to synaptic deterioration and cognitive decline.

Classical carbonyl scavengers, such as carnosine and aminoguanidine, have been widely explored as therapeutic strategies to attenuate carbonyl stress through direct chemical neutralization of reactive dicarbonyl species, including MGO. By limiting AGE formation and glycation-associated macromolecular damage, these agents effectively reduce carbonyl burden at the chemical level (Davies and Zhang, 2017; Schalkwijk and Stehouwer, 2020). However, their protective actions are largely confined to antiglycation and detoxification processes, with limited evidence supporting active interference with carbonyl stress-mediated inflammatory signaling pathways in AD (Sun et al., 2016; Zhang et al., 2021).

Given the complexity of the underlying mechanisms of AD, therapeutic efficacy cannot be fully assessed based on a single parameter such as direct carbonyl scavenging activity. Importantly, MGO-derived AGEs function not only as glycation end products but also as potent ligands that amplify RAGE-dependent inflammatory signaling, thereby linking carbonyl stress to neuroinflammation and synaptic dysfunction (Lai et al., 2022; Lin et al., 2025). Traditionally, therapeutic approaches targeting carbonyl stress have primarily focused on direct carbonyl scavenging, antioxidant-mediated reduction of reactive carbonyl formation, or modulation of enzymes involved in MGO detoxification. In contrast, the present study suggests that fucoxanthin may represent a potentially distinct approach by simultaneously reducing MGO accumulation and attenuating RAGE/NF-κB-related inflammatory responses, thereby linking carbonyl stress with inflammatory amplification.

The present findings are further supported by evidence from human AD brain tissue, where elevated RAGE expression and enhanced Aβ-RAGE interactions are closely linked to microglial activation and synaptic dysfunction (Yan, 2012; Dong et al., 2022). Consistent with this, the present study demonstrated that Aβ exposure induces MGO accumulation, which subsequently upregulates RAGE expression, a key initiating event in the carbonyl stress pathway. Fucoxanthin treatment markedly reduced MGO accumulation in Aβ_1-42_-injected mice, being associated with reduced RAGE and NF-κB-related inflammatory responses. These findings suggest that fucoxanthin may contribute to reducing the amplification cycle between carbonyl stress and neuroinflammation. Furthermore, since MGO readily forms irreversible adducts with proteins and nucleic acids, lowering its levels contributes to the preservation of structural and functional cellular integrity (Fuloria et al., 2020).

Given that RAGE activation is a primary upstream regulator of NF-κB signaling, fucoxanthin treatment was accompanied by reduced RAGE expression and NF-κB activation markers in Aβ-treated cells. This effect was further validated in the Aβ_1-42_-injected mice, where oral fucoxanthin administration (100 or 200 mg/kg) effectively inhibited hippocampal NF-κB activation and its downstream inflammatory mediators, including COX-2, iNOS, IL-1β, and TNF-α. Comparable anti-inflammatory effects have been reported for sulforaphane, a well-known anti-inflammatory compound from cruciferous vegetables, which reduced the expression of IL-1β and TNF-α via inhibiting translocation of NF-κB p65 at 5 μM in an Aβ_1-42_-treated human macrophage cell (Jhang et al., 2018). In an Aβ_1-42_-treated human macrophage cell model, sulforaphane was reported to reduce IL-1β and TNF-α expression by inhibiting NF-κB p65 nuclear translocation. Several marine-derived compounds, including phlorotannins, fucoidan, and astaxanthin, have been reported to attenuate inflammatory signaling primarily through modulation of NF-κB and MAPK pathways (Cho et al., 2023; Jayasinghe et al., 2023; Liyanage et al., 2023; Jung et al., 2024; Wu et al., 2024). Compared with these agents, fucoxanthin additionally reduced systemic methylglyoxal accumulation, suggesting potential involvement in carbonyl stress-related inflammatory regulation. Microglial activation is a well-established feature of Aβ-induced neuroinflammation, and has been shown to be amplified by the upregulation of RAGE-positive microglia. In transgenic AD models, RAGE-Aβ interactions initiate signal transduction cascades and promote cytokine production, further contributing to microglial recruitment and activation (Fang et al., 2010). Consistent with these mechanisms, the Iba-1 immunostaining results demonstrated that fucoxanthin treatment significantly reduced Iba-1-positive microglial density in hippocampal subregions. These regions, though functionally distinct, collectively support memory-related processes such as pattern completion (CA3), temporal encoding (CA1), and pattern separation (DG) (Kesner, 2013). The reduction in Iba-1 immunoreactivity indicates attenuation of microglial activation, particularly in CA1 and CA3 where Aβ-induced microglial activation was confirmed. This interpretation is supported by prior studies showing that reduced hippocampal microglial activation is associated with attenuation of neuroinflammatory pathology in AD models (Ren et al., 2024).

The hippocampus is one of the brain regions most vulnerable to Aβ-associated pathology in AD (Christensen et al., 2008; Forner et al., 2017; Ray et al., 2024). Therefore, the present study focused on hippocampal inflammatory and synaptic responses to evaluate the neuroprotective effects of fucoxanthin. Beyond its anti-inflammatory effects, fucoxanthin significantly restored PSD-95 expression in Aβ_1-42_-injected mice, suggesting its role in protecting synaptic integrity. PSD-95 is a critical scaffolding protein at excitatory synapses, essential for maintaining synaptic architecture and functional plasticity (Dore et al., 2021), and its downregulation is closely associated with impaired long-term potentiation and memory deficits in AD models (Zhao et al., 2019). Given that sustained carbonyl stress and MGO accumulation are increasingly recognized as key contributors to synaptic deterioration in AD, the observed recovery of PSD-95 expression is consistent with the attenuation of carbonyl stress-driven pathological signaling demonstrated in the present study. Thus, restoration of PSD-95 may reflect a downstream synaptic outcome of MGO/RAGE/NF-κB axis suppression rather than an isolated molecular event, further supporting the role of carbonyl stress regulation in preserving synaptic function under Aβ-induced conditions (Nigro et al., 2019; Yang et al., 2022). In parallel, fucoxanthin significantly increased synaptophysin expression despite the absence of a marked reduction after Aβ_1-42_ injection, indicating a direct modulatory effect on presynaptic stability rather than a simple reversal of Aβ-induced loss. This differential pattern between postsynaptic and presynaptic markers is consistent with previous reports indicating that postsynaptic structures are more susceptible to the early stages of Aβ-induced pathology, whereas presynaptic components appear relatively unaffected, though responsive to neuroprotective interventions (Gylys et al., 2004).

The selected doses (100 and 200 mg/kg) were based on previous studies in which fucoxanthin was orally administered to animal models within a dose range of 50 to 200 mg/kg (Lin et al., 2016; Zhang et al., 2017; Zhang et al., 2025; Chen et al., 2023; Anand et al., 2024; Jiang S. et al., 2025; Jiang Y. et al., 2025; Lee et al., 2025). Moreover, previous toxicological studies have demonstrated that fucoxanthin produces no mortality, no histopathological organ damage, and no alterations in hematological or serum biochemical parameters even after repeated oral administration, with a reported no-observed-adverse-effect level (NOAEL) of 200 mg/kg/day and an LD_50_ exceeding 2,000 mg/kg in rodents (Beppu et al., 2009; Iio et al., 2011; Peng et al., 2011).

Although direct pharmacokinetic and brain distribution analyses were not performed in the present study, previous studies have reported that orally administered fucoxanthin is metabolized into fucoxanthinol, which is absorbed into systemic circulation and distributed to multiple tissues *in vivo* (Hashimoto et al., 2009; Hashimoto et al., 2012; Takatani et al., 2023). Recent studies further demonstrated that fucoxanthinol exhibits permeability across blood–brain barrier models and that orally administered fucoxanthin can be detected in brain tissue following supplementation (Cokdinleyen et al., 2025; Park et al., 2025). These findings support the potential brain availability of fucoxanthin-derived metabolites under *in vivo* conditions.

Since AD is a multifactorial and slowly progressive neurodegenerative disease, no single experimental model fully recapitulates all aspects of human AD pathology (Akhtar et al., 2022; Dhapola et al., 2023). The intracerebroventricular Aβ_1-42_ injection model used in the present study enables controlled induction of acute amyloid-associated carbonyl stress and neuroinflammatory changes within a relatively short experimental period, making it suitable for evaluating the early molecular effects of fucoxanthin. However, this model does not fully reproduce the chronic neurodegenerative progression and long-term cognitive impairment characteristic of human AD. Accordingly, additional studies using chronic and genetically driven AD models with behavioral and cognitive assessments will be important for further evaluating the long-term and functional relevance of the present findings.

Taken together, these findings suggest that fucoxanthin exerts neuroprotective effects against Aβ-induced pathology, accompanied by reductions in carbonyl stress-related inflammatory signaling. Compared with conventional carbonyl scavengers that primarily focus on detoxification, fucoxanthin was associated with reduced MGO accumulation, decreased RAGE/NF-κB-related inflammatory responses, and preservation of synaptic markers. The concurrent reduction in MGO levels, inflammatory mediators, and microglial activation, together with restoration of PSD-95 expression, further supports a role for carbonyl stress-associated pathways in the neuroprotective actions of fucoxanthin. These findings highlight fucoxanthin as a promising candidate for mitigating Aβ-induced neuroinflammatory and synaptic alterations.

## 5 Conclusion

The present study suggests that fucoxanthin confers neuroprotective effects against Aβ-induced pathology by modulating carbonyl stress-associated inflammatory and synaptic alterations. Fucoxanthin treatment was accompanied by reductions in MGO accumulation and RAGE/NF-κB-related inflammatory signaling. The compound also preserved synaptic integrity, as indicated by restored expression of PSD-95 and synaptophysin *in vivo*. This focused mode of action supports its relevance as a dietary candidate targeting early-stage AD-related pathological processes. These observations highlight the potential relevance of fucoxanthin as a dietary candidate targeting early-stage AD-related pathological processes.

## Data Availability

The raw data supporting the conclusions of this article will be made available by the authors, without undue reservation.
