# Correction: Fucoxanthin attenuates carbonyl stress and neuroinflammation by modulating MGO/RAGE/NF-κB axis in Aβ-induced models

**DOI:** 10.3389/fphar.2026.2023015

**Published:** 2026-10-29

**Authors:** Nayoung Lee, Kumju Youn, Huiyoung Kwon, Dong Hyun Kim, Chi-Tang Ho, Mira Jun

**Affiliations:** 1 Department of Health Sciences, The Graduate School of Dong-A University, Busan, Republic of Korea; 2 Department of Food Science and Nutrition, Dong-A University, Busan, Republic of Korea; 3 Center for Food and Bio Innovation, DAU G-LAMP Project Group, Dong-A University, Busan, Republic of Korea; 4 Department of Pharmacology, School of Medicine, Konkuk University, Seoul, Republic of Korea; 5 Department of Advanced Translational Medicine, School of Medicine, Konkuk University, Seoul, Republic of Korea; 6 Department of Food Science, Rutgers University, New Brunswick, NJ, United States

**Keywords:** Alzheimer’s disease, amyloid beta, carbonyl stress, fucoxanthin, MGO/RAGE/NF-κB axis, neuroinflammation

The funder Global - Learning & Academic research institution for Master’s·PhD students, and Postdocs (LAMP) Program of the National Research Foundation of Korea (NRF) grant funded by the Ministry of Education, RS-2025-25440216 to Mira Jun and Kumju Youn, was erroneously omitted.

The original version of this article has been updated.

